# Measuring Food Anticipation in Mice

**DOI:** 10.3390/clockssleep1010007

**Published:** 2018-10-26

**Authors:** Tomaz Martini, Jürgen A. Ripperger, Urs Albrecht

**Affiliations:** Department of Biology, Unit of Biochemistry, University of Fribourg, 1700 Fribourg, Switzerland

**Keywords:** food anticipation, circadian rhythms, chronobiology, peripheral oscillators, mouse activity, wheel running, *Period2* (*Per2*), metabolic disease, feeding, food-entrainable oscillator (FEO)

## Abstract

The interplay between the circadian system and metabolism may give animals an evolutionary advantage by allowing them to anticipate food availability at specific times of the day. Physiological adaptation to feeding time allows investigation of animal parameters and comparison of food anticipation between groups of animals with genetic alterations and/or post pharmacological intervention. Such an approach is vital for understanding gene function and mechanisms underlying the temporal patterns of both food anticipation and feeding. Exploring these mechanisms will allow better understanding of metabolic disorders and might reveal potential new targets for pharmacological intervention. Changes that can be easily monitored and that represent food anticipation on the level of the whole organism are a temporarily restricted increase of activity and internal body temperature.

## 1. Introduction

The circadian (circa—lat. approximately; dies—lat. day) timing system is a network of brain clocks and peripheral oscillators that enable mammals to adapt to recurring daily events and environmental changes, such as light/dark phases [1,2,3]. These timing systems in tissues are either entrained by light through the master pacemaker, the suprachiasmatic nucleus, or by other environmental cues, such as food, in which case peripheral oscillators might be uncoupled from the central pacemaker [2,4,5,6,7,8]. The food-anticipatory activity is an example of such uncoupling of behaviour from the circadian routine, because it produces a food-seeking response of animals due to food availability rather than entrainment by light [4,6,7,9]. Recent experiments by our laboratory have shown that the liver transmits a food-anticipatory signal to the brain, the liver-derived ketone bodies, which, together with a pre-prandial increase in internal body temperature and a shift of the peripheral clocks, may indicate that the food-entrainable oscillators are of systemic nature [4,6].

**Figure 1:**
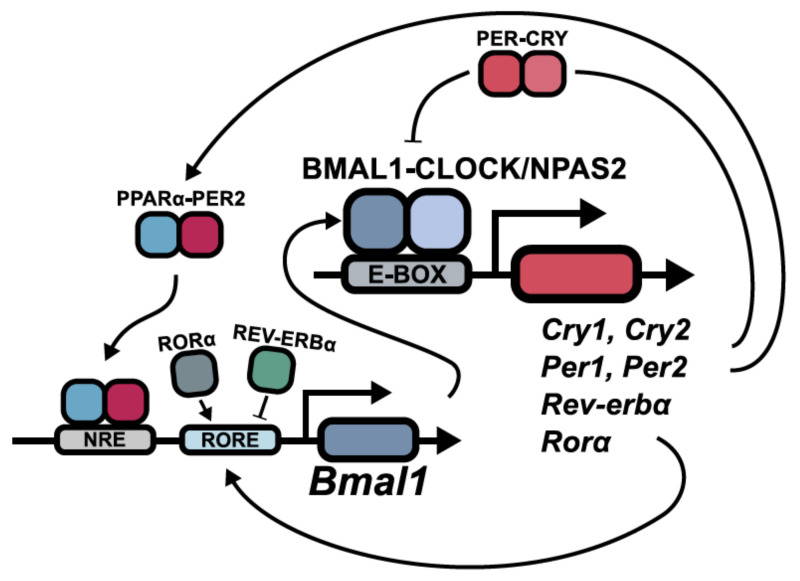
Schematic representation of the molecular clock. The transactivational complex BMAL1-CLOCK activates genes with E-BOX regulatory sites. PER-CRY complexes reduce this transactivational activity of BMAL1-CLOCK and provide a negative feedback. However, PER2 can also interact with nuclear receptors, for example, PPARα (peroxisome proliferator-activated receptor α) and positively regulate *Bmal1* through activation of nuclear receptor response elements (NRE). On the level of transcriptional regulation of *Bmal1*, there is also competition between RORα and REV-ERBα, providing either transcriptional activation or repression, respectively, by binding to ROR response elements (RORE) and thereby stabilizing the molecular clock [1,10,21,22].

At the molecular level, circadian rhythms are generated by a set of clock genes and their auto-regulation through transcriptional and translational feedback loops. The positive factors BMAL1 (brain and muscle ARNT-like protein 1; also ARNTL—aryl hydrocarbon receptor nuclear translocator-like protein 1) and CLOCK/NPAS2 (circadian locomotor output cycles kaput, neuronal PAS domain protein 2) bind as heterodimers to E-box motifs present in target genes, for example, *Per* (*Period*; homologues *1*, *2*, *3*), *Cry* (*Cryptochrome*; homologues *1*, *2*), *Ror* (*retinoic acid receptor-related orphan receptor*) and *Rev-Erb* (*Reverse c-ErbAα* [the opposite strand of thyroid receptor α]). These in turn either positively or negatively regulate *Bmal1* and *Clock*/*Npas2*, providing a feedback loop (Figure 1) [2,3,8,10].

The molecular clock has gathered interest recently, as its multiple outputs are involved in regulation of some of the most important functions of organisms [11]. Defects in the system can lead to metabolic disease, neurological disorders and cancer [12,13,14,15,16,17]. Therefore, it is vital to have an in-depth understanding of how the molecular clock can affect these systems.

Development of robust behavioural experiments allows us to compare different groups of genetically manipulated animals to understand the roles of specific genes [18]. In case of metabolism, unravelling gene function and describing metabolic signalling provides advances in understanding metabolic regulation, processes that lead to feeding and metabolic adaptation. This, in turn, allows interpretation of possible pathophysiological states or even provides potential new targets for pharmacological intervention. There are limited resources available to tackle and adjust food intake itself as the source of a potential problem.

**Figure 2:**
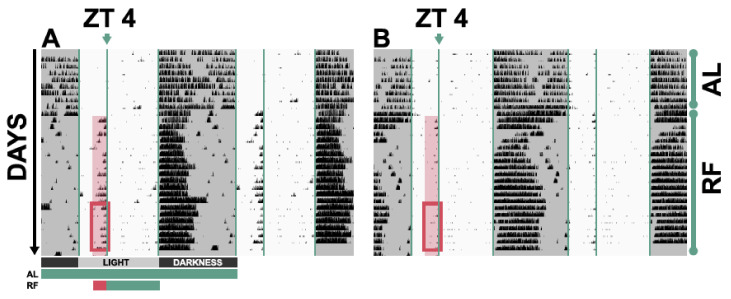
Representative results of restricted feeding experiments shown as double-plotted actograms, in which column height over time shows the activity of an animal. Two days are represented in each row, with the day on the right being repeated in the following row. Times of day when lights were off are shaded in grey and food-anticipatory activity during ZT 2–4 is highlighted, with additional emphasis on the last week of restricted feeding, visible within a rectangle. The green and red bars under the actograms represent time of analysis and food availability, like in Figure 3. AL—ad libitum; RF—restricted feeding. (**A**) A control animal shows food-anticipatory activity before food availability at ZT 4. (**B**) A genetically modified animal shows strongly reduced food-anticipatory activity, suggesting an involvement of the missing gene in anticipation of food.

Mice are nocturnal animals, meaning they are active during the dark period, when they also eat under regular conditions. Repeatedly restricting their access to food to a predetermined period with light and reducing the amount of food available will change their behaviour, as they need to adapt to new conditions [7,19,20]. This adaptation, shown in different activity and internal temperature patterns, generally described as food anticipation, can be studied with wheel-running experiments and telemetrics [6,19]. Mice start showing food anticipation about two hours before feeding, so an interval starting two hours before feeding and until feeding can be analysed for changes in activity (Figure 2) and internal body temperature. The induction of such behavioural changes can be exploited in multiple ways: (1) Determination of the players involved in molecular signalling, for example, with loss-of-function studies, (2) Studying temporal patterns of physiological and molecular changes, or (3) Restoring the lost behaviour by rescue experiments. These approaches can be done in a tissue-specific manner. Controlled lighting conditions (LD conditions), which are necessary for such experiments, are usually described using zeitgeber time (ZT), with ZT 0 being lights on and ZT 12 being lights off. A light source that covers the whole spectrum and mimics natural light is required. Food-anticipatory experiments described in our protocol are performed with 12 h periods of light followed by 12 h periods of darkness, resulting in a 12 h light, 12 h dark cycle (LD 12:12).

**Figure 3:**
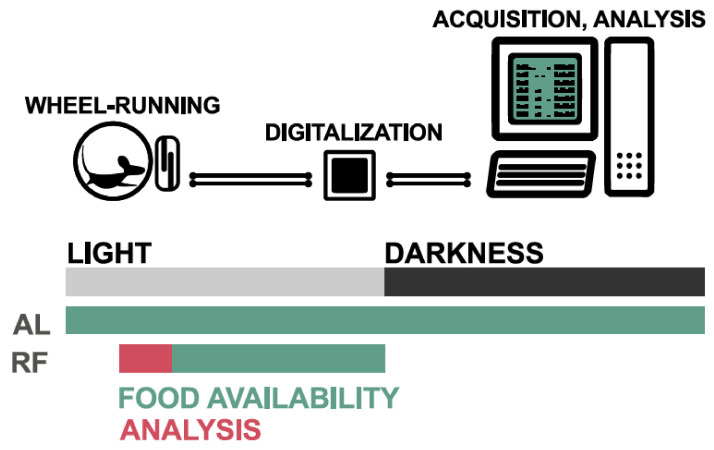
Schematic representation of the experimental setup for restricted feeding. Mice are subjected to 12 h light, 12 h dark cycles. First, the mice are adapted to the setup, then subjected to temporal (ZT 4–12) and caloric food restriction (80% of daily intake for the first week and 70% for the following two weeks). The wheels in cages are coupled to a magnet that, for each revolution, closes the circuit in a connector located just next to the axis. This creates binary data that can be digitalized and processed.

We perform wheel-running experiments using custom built cages according to local legislation on animal experimentation. The data is digitalized using a USB interface from Actimetrics and the wheel revolutions are monitored and processed using the ClockLab software (Figure 3). Telemetrics is performed with the VitalView system that measures general mouse activity as a number of signal strength changes and internal body temperature using a thermistor, which controls an oscillator circuit whose frequency is related to the thermistor temperature. The system is wireless and rechargeable. Hence, it is possible to monitor behavioural and physiological changes in free-running animals.

## 2. Protocols

### 2.1. Restricted Feeding Protocol

1. Place min. 3-month-old experimental and control animals into individual cages and give them ad libitum access to standard chow (free access to unlimited amount of food).
2. On the 7th, 14th and 21st day clean cages and measure mouse body mass and mass of food eaten.
3. On the 21st day remove food just before lights off (ZT 12).
4. Use the gathered data to calculate caloric requirements of individual mice. If there are slight differences in the weekly amount of food eaten during adaptation, the values of the final week should be used to determine the regular daily intake.
5. For the following 3 weeks, starting on the 22nd day, challenge mice by limiting food access from ZT 4 to ZT 12 and by providing 80% of their regular daily intake in the first week and 70% of their regular intake in the following two weeks. Prepare portions of food in advance, so that time with animals is minimised. The body mass of animals should never drop below 80% of the mass they had in the third week of ad libitum feeding. In such an event, remove the mice from the experiment and house them with ad libitum access to food to recover.
6. Analyse and compare activities and/or internal body temperature profiles of animals during the last week of ad libitum conditions: the activity levels and temperature profiles should be comparable.
7. Analyse and compare activities and patterns of internal body temperature during ZT 2–4 of the last week of restricted feeding conditions.

### 2.2. Monitoring Activity Using Wheel-Running Cages

Perform wheel-running experiments according to Jud et al.: A guideline for analyzing circadian wheel-running behavior in rodents under different lighting conditions [23].

1. Set up a system for wheel rotation monitoring.
2. Validate wheel revolution count.
3. Place the mice into wheel-running cages. Note: Special attention should be given to the amount of bedding and nesting material. Too much bedding and nesting will result in partial or total wheel blockage, which may produce false measurements.
4. Perform restricted feeding experiment. Record wheel-running activity during the adaptation under ad libitum feeding as well as during the restricted feeding.
5. Change cages once a week when weighing mice and food, just before lights off, so that disturbance of mice and their natural rhythms is minimal. If additional inspection of animals is absolutely necessary, do it just before lights off or during the beginning of the activity period of mice using night vision goggles.
6. Export and analyse the data.

### 2.3. Surgical Implantation of Telemetric Transponders

1. Prepare necessary medication using good laboratory practice to avoid post-surgical complications and potential abnormal behaviour due to infection and/or inflammation. Prepare all medication in a biosafety cabinet under aseptic conditions. We recommend using 0.9% NaCl infundibile for dilution of the medication rather than in-lab prepared sterile 0.9% NaCl which is not apyrogenic. We also suggest preparing the medication into apyrogenic vials that need to have the rubber stopper disinfected with alcohol. Prior to recovery of fluid from a vial, the syringe should contain a volume of air that will match and replace the volume of liquid required. Likewise, for injection into an empty vial, the plunger of the syringe should be released before the needle and syringe are removed from the vial, to allow removal of displaced air and adjustment of pressure. The liquid-air exchange can be done in multiple small steps by manipulating the plunger.
  1.1. Anaesthetic: 2.4 mg ketamine hydrochloride and 0.01 mg medetomidine hydrochloride per 30 g mouse (80 mg/kg, 0.3 mg/kg). Usually, ketamine hydrochloride is available at 100 mg/mL and medetomidine hydrochloride is available at 1 mg/mL concentration. The appropriate anaesthetic can be prepared by taking 0.120 mL ketamine and diluting it in 0.9% NaCl to a total volume of 1 mL and by taking 0.100 mL medetomidine and diluting it in 0.9% NaCl to a total volume of 1 mL; mix the ketamine and medetomidine diluted solutions in a 2:1 ratio to get the anaesthetic that can be injected as 0.300 mL per 30 g mouse (and adjusted to mouse body mass) intraperitoneally [24].
  1.2. Painkiller: 0.15–0.30 mg carprofen per 30 g mouse (5–10 mg/kg). Usually, carprofen is available at 50 mg/mL concentration. The appropriate painkiller can be prepared by taking 0.050 mL carprofen and diluting it in 0.9% NaCl to a total volume of 5 mL. This solution can be injected as 0.300–0.600 mL per 30 g mouse (and adjusted to mouse body mass) subcutaneously. Injecting the bolus is also a way to provide fluid.
  1.3. Anti-sedation: 0.03 mg atipamezole hydrochloride per 30 g mouse. Usually, atipamezole hydrochloride is available at 5 mg/mL concentration. The appropriate anti-sedation can be prepared by taking 0.040 mL atipamezole hydrochloride and diluting it in 0.9% NaCl to 0.800 mL. This solution can be injected as 0.120 mL per 30 g mouse (and adjusted to mouse body mass) intraperitoneally.
2. Clean and sterilize surgical tools and implant. Surgical tools can be sterilized by heat or with a liquid sterilizing agent. The implants can usually be sterilized using liquid sterilizing agents, such as benzalkonium chloride. Consult the manufacturer’s manual. Before use, wash implants with 0.9% NaCl infundibile.
3. Weigh the mouse and calculate required doses of medication.
4. If you are using an implant that can be fixed into place with stitching material, attach the stitching material to the transponder.
5. Immobilize the mouse by grasping the skin fold at the rear of its neck and holding its tail. Manoeuvre it into a head-down position, so that the intestines of the mouse move towards the upper part of the abdominal cavity. This will allow intraperitoneal injection of medication with the intestines being out of the way of the needle.
6. Intraperitoneally inject mouse with anaesthetic according to institutional rules and animal experimentation legislation. Place the mouse in its cage and wait for it to become unresponsive. Then roughly clean the mouse of bedding and place it onto an aseptic and heated (42 °C) working surface. Disinfect the belly, for example, with povidone-iodine. Check depth of anaesthesia by pinching skin between toes. Avoid pinching the toe itself as this can cause injury and pain after surgery.
7. Apply hydrogel to eyes to prevent drying out.
8. Make a vertical incision of a length of 2 cm that stops around 1 cm below the rib cage. Skin can be shaved before the surgery.
9. Put the chip into the abdominal cavity, not too close to the skin to avoid false measurements and fasten it in place according to manufacturer’s instructions. Pull the stitching material out through the wound, so it can be fastened into place when doing the suture.
10. Close the wound; we use a simple interrupted suture with 3–5 stitches.
11. Apply non-steroid anti-inflammatory medication subcutaneously. Apply anti-sedation intraperitoneally (see 5).
12. Put the mouse into a cage on a heating mat, place food inside of the cage for easy access. Food can be pre-soaked in water. Nesting material soaked in water (or a piece of apple) can be placed close to the food so the mouse can chew on it. The recovery cage should not have bedding as this could go into the airway of the mouse.
13. Monitor mouse at least every 30 min until fully awake and mobile. If necessary, re-apply hydrogel to eyes. Place the mouse into its home cage. Allow at least 3–5 days of recovery before recording.
14. After the implantation of transponders and recovery, proceed to the experimental protocol. After surgery, perform ad libitum feeding for 3 weeks in order to monitor potentially abnormal behaviour (activity patterns, temperature fluctuations) and to establish average daily temperature rhythms and regular food intake. Determine the regular food intake when the implant is in place, because the abdominal implant’s physical volume may affect the amount of food eaten.
15. Perform the restricted feeding experiment and record internal body temperature and activity with the telemetrics system. Analyse the data.

## 3. Discussion

The protocol describes one of the approaches that are used by different research groups to study the adaptation to daytime feeding, seen as an uncoupling of feeding-related behaviour and behaviour that is a consequence of entrainment to the light-dark cycle. Food availability from ZT 4 to ZT 12 is not a general consensus and it was chosen to maximize the window of food availability during the light phase. Furthermore, such a protocol has been used to evaluate multiple animal genotypes and it enables comparison of experimental results. The obtainable results are easily reproducible and consistent with publications on the involvement of molecular clock genes in food anticipation [18,19,25]. In our case, the procedure involves using reduced daily portions of food to prevent potential over-eating during the light phase, while at the same time ensuring enough nourishment for the animals. The mice are first provided with a slightly higher amount of food in the first week to allow for gradual adaptation. Such an approach therefore guarantees that the mice will be hungry before feeding time and that the hunger or homeostatic mechanisms will be involved in food-seeking behaviour. However, this “safety measure” is not necessary for exploration of physiological and behavioural changes before daytime feeding and some research groups do not employ it or they simply use a shorter window of daytime food availability.

It is worth noting that animals need to be 3 months old, because at this age the body mass stabilizes. The body mass of animals during adaptation should stay more or less constant. Body mass fluctuation might suggest animals are too young or indicate an underlying health problem. On the other hand, mice that are too old begin to gradually show a decrease in wheel-running activity.

The first three weeks of ad libitum feeding are necessary for adaptation of the mice to the new environment, measurements of caloric intake under ad libitum conditions and as a negative control for potential differences in activity between groups of animals. For wheel-running experiments, the adaptation period may be extended if mice do not show adequate wheel-running entrainment. The entrainment means that the mice, under regular conditions, begin running when the lights are switched off and stop running at the onset of light.

The feeding should be precise so that the quantification (ZT 2–4) does not encompass the period when the feeding was performed. Animals might respond to presence of an experimentalist handling food. Therefore, we suggest giving the food at exactly 5 min after ZT 4 and doing subsequent quantification exactly up to ZT 4.

While temperature measurements are straightforward, one should distinguish different types of activity, as there is a big difference between general activity (e.g., scratching and grooming) and voluntary activity on the wheel, which also presents a motivational aspect of behaviour. To turn the wheel, the mouse has to be fully awake and probably also has to have an appropriate muscle tone.

The food-anticipatory activity can usually be monitored simultaneously as temperature using telemetrics. However, such monitoring of activity describes general activity and encompasses random movements in addition to voluntary activity. For this reason, activity monitoring using telemetrics often results in noisier measurements leading to lower sensitivity. Furthermore, because in our case the activity measured with the VitalView telemetrics system is correlated to changes in signal strength, the measure of movement is purely qualitative, as stated by the manufacturers and cannot be used for reliable comparison between groups [26]. However, it does give a quick overview of animals being active or inactive during the period of potential food anticipation. Additionally, monitoring wheel-running activity is non-invasive, which makes it the preferable approach. General guidelines for wheel-running experimentation that comprise the current knowledge of monitoring clock-regulated animal behaviour allow objective and comparable experimental procedures [23]. There are other approaches to assess the activity and temperature that different research groups employ. An alternative to monitoring wheel running is tracking activity by beam breaks in cages with emitters and receivers of infrared light. The activity is related to the number of signal interruptions. This too is a non-invasive approach. Usually, horizontal movement (x and y) is quantified as movement of mice [27,28].

Even though the described approaches are robust, noise insulation, minimal access by experimental personnel, white noise generation and ventilation is necessary to avoid random environmental effects on the experiment. Furthermore, the correct ambient temperature is of great importance and a drop in temperature might result in dramatically decreased activity during the dark phase, especially under the restricted feeding regime, further supporting the importance of a baseline ad libitum recording.

Wheel running is usually represented by actograms (Figure 2), where column height over time represents activity of an animal, with multiple days plotted one under the other. Actograms usually represent relative changes in activity of a single mouse and cannot be directly visually compared. Also, the actogram representation may be approximated due to technical constraints and does not represent the full resolution of recording: in our case, the actogram representation is rounded to a pixel; for example, on a full HD screen we managed to get a max. resolution of ±3 min and even lower resolutions can be expected in some representations. These resolutions can then be replicated when exporting the graphical representations of activity. This often means that such representation could lead to false conclusions. A different representation and subsequent analysis of exported data is therefore necessary. We suggest making average daily activity profiles of required resolution for the last week of ad libitum feeding and last week of restricted feeding for each mouse. The last week of ad libitum feeding represents a negative control and the last week of restricted feeding is the experiment. In short, such a representation is done by taking activity values of an individual mouse for each time-point for the required number of days (e.g., 7 days). These activities are then averaged so that one gets an average activity of the mouse at a specific time. Such representations of daily activity over time are known as activity profiles. Since they take into consideration a longer period of time, potential one-time observations (that could lead to false conclusions) are masked. Several representations like this, with various genotypes, may be seen in papers of Feillet et al., 2006, Chavan et al., 2016, Mistlberger, 2009 and Mendoza, 2010 [4,9,19,25]. This approach provides a good way of studying the activity patterns of mice with normal food anticipation versus those with difficulty in anticipating food [4,25]. As the previously published data goes beyond the scope of this protocol paper, we strongly encourage the reader to look at the graphical representations and acquire a feeling for what kind of activity data one should expect.

It is worth stressing the importance of visually inspecting activity profiles using an absolute (not a relative) scale. Visual inspection should show no activity of animals during ZT 2–4 under ad libitum conditions and an activity peak under restricted feeding. Activity under ad libitum feeding at ZT 2–4 might suggest disturbances in the experimental facility. Visually inspecting each animal’s profile will also allow seeing potential other abnormalities.

When visualising data from telemetrics, similar approaches and comparisons between groups can be applied. However, the transfer of data is wireless, which might mean that some data points are missing or invalid. Because of the high frequency of data acquisition, the few invalid data points can be easily dismissed. In our case, the VitalView Data Acquisition System’s software is used to perform an activity filter on 1 min bins of general activity to remove non-physiological values from the data set and to smooth high-sensitivity data. An invalid points filter is used to replace invalid or missing data points with the last valid data sample of 1 min temperature bins. Like with the wheel-running data exports, profiles showing either activity or temperature over time can be created.

The abdominal temperature profiles of mice with ad libitum access to food show higher temperature during the dark period and a lower body temperature during the light period. In restricted feeding experiments, there is an increase in the temperature about 2 h before feeding time in groups of animals that anticipate food. The animals that do not anticipate food also show an increase in body temperature, but only after food has been given to them. In this case the rise in body temperature is a consequence of food intake. Therefore, the difference is not only visible in the amplitude but also (or even more so) in the onset of the increase in body temperature. The increases are relative and highly dependent on ambient temperature and the genetic background of mice and probably also their body mass that determines the surface area to body volume ratio. Therefore, the experimental design requires reducing the number of variables to a minimum and performing the experiment in the same conditions and at the same time. Caution should be taken when pooling data from consecutive experiments. Importantly, in either case, whether animals anticipate food or not, daytime feeding dramatically reduces the abdominal temperature at the end of the night period, which is a common observation [6]. Representative temperature profiles can be studied with the help of previously mentioned papers.

Analysis of the exported and visualised data is necessary. Food-anticipatory activity can be analysed as total activity (e.g., average wheel revolutions per min) during ZT 2–4. Two groups of animals can be compared using a t-test, while multiple groups can be compared using ANOVA and post-hoc tests. This approach of comparing activity in a predefined time-frame considers exploration of the amplitude of the food-anticipatory activity, because the total activity is merely a sum of signals (wheel revolutions). This activity may be compared to total activity during the night period. A potential difference in activity between groups can also be explored by comparing the activity over time (comparing curves) by using a two-way ANOVA, where the two independent variables represent the genotype and time, or a generalized linear model. The approaches described for curve comparison can also be used to compare internal body temperature patterns between groups. A curve comparison provides a good overview of behaviour of a group of animals, because it describes both the amplitude(s) of changes due to food anticipation (in relation to activity or temperature in the dark period) and also potential differences in onset of these changes in comparison to controls, while standard errors at specific times show variability within a group. We analyse the differences in food anticipation as differences during the window from ZT 2 to ZT 4. This allows comparison of newly obtained results with available data [25]. One might, however, argue that observations of different patterns of activity than ours or different variations of the protocol might need adaptation of the analysis period. Indeed, even in our protocol, individual mice may start running before ZT 2. However, the average group activity generally fits this analysis window. In any case, group activity profiles clearly show the running pattern. Care needs to be taken when analysing prior activity or continuous activity from the dark period; they may resemble food-anticipatory activity but might have a different origin. Sometimes it is difficult to distinguish food-anticipatory activity from the spontaneous running one might observe in animals that intensively and desperately seek food in cages without chow. Disturbances in the facility might also arouse mice and such activity has nothing to do with the anticipation of food.

Isolating feeding from the usual activity period may provide insights on general metabolic regulation and/or the motivational aspects of food-seeking behaviour. However, first, resolving food anticipation itself might be necessary. The origins of food anticipation have appeared elusive for a long time, with the scientific community often not having a consensus over the tissues and mechanisms underlying the adaptation to daytime feeding [29,30]. In recent years, however, there have been advances that helped to at least partially clarify the underlying causes of the observed pre-prandial changes [4,27,28]. While the scientific community still approaches the idea of a systemic nature of the phenomenon with caution, there is emerging proof that not only the central nervous system is involved in this behaviour but also peripheral tissues.

Undeniably, there might be different signalling pathways that lead to similar observations of a phenotype. In this respect, the persistence of signals from the periphery in form of ketone bodies, which are necessary for initiation of food anticipation, may be further explored. It is clear, that these signals are at least necessary for induction of the mechanisms that are expressed as repetitive daily manifestations of physiological adaptation.

Furthermore, it would be unwise to neglect the potential of learning processes that might work hand in hand with the metabolic signalling that leads to food anticipation in the first place. An excellent example of such an observation was provided by Tan, Knight and Friedman, where mice with *Agrp*-driven neuron ablation could initially not adapt to restricted feeding for multiple days, even experiencing mortality, but later started to show an increase in activity before feeding. Interestingly, these mice recover the anticipatory activity after about 10 days. Perhaps this might be a memory-related observation or an adaptation utilising an alternative mechanism. In either case, the experiment shows that there are different paths that might lead to pre-prandial activity [27].

The described methodology gives researchers a straightforward and robust approach to explore mechanisms that lead to food anticipation. With increasing metabolic disease prevalence, it is extremely important to explore not only metabolism after feeding but also behaviour that leads to food anticipation and the motivational aspects of eating. Perhaps not food itself but rather hunger and short-term starvation lead to described changes in activity and body temperature, as suggested by the requirement of liver-derived ketone body signalling in food anticipation. This opens up a completely new perspective on signalling before feeding and raises questions regarding its temporal patterns, especially when trying to compare healthy individuals with those that suffer from various metabolic illnesses.
